# Supplementary material for: Generalized Probabilistic Approximate Optimization Algorithm
Source: Nat Commun. 2025 Dec 8;17:498. doi: 10.1038/s41467-025-67187-5 (PMC12804969; doi:10.1038/s41467-025-67187-5)
Supplement: Supplementary file 1 — Supplementary Information [file 41467_2025_67187_MOESM1_ESM.pdf]

Supplementary Information

Generalized Probabilistic Approximate Optimization Algorithm

Abdelrahman S. Abdelrahman, Shuvro Chowdhury, Flaviano Morone and Kerem Y. Camsari

In the main manuscript, we discussed privatized-beta-based PAOA, and global-beta-based PAOA. Here, we present the implementation of fully parameterized PAOA (See Algorithm.S1), which is used in learning the full-adder target states by minimizing the negative log-likelihood cost (expressed in equation (S.4)), see Section. 1. The exact same structure of the algorithm is also used for learning the majority gate correct states, however, with private β as ansatz, see Section. 2. The

Algorithm S1: PAOA: fully-parameterized

Input : number of nodes N, number of layers p, number of experiments N_E, initial variational parameters $(J^{(1)}, J^{(2)}, \ldots, J^{(p)})$, tolerance $\varepsilon_{\text{step}}$, maximum iterations $t_{\max}$, truth table

Output: trained set of weights $(J^{(1)}_{\text{opt}}, J^{(2)}_{\text{opt}}, \ldots, J^{(p)}_{\text{opt}})$

Function `p-computer` (J_{init}, N, p) **:**
- initialize all spins randomly
- **for** $i \leftarrow 1$ **to** p **do**
  - $J \leftarrow J^{(i)}$
  - **for** $j \leftarrow 1$ **to** N **do**
    - solve equations (S.1) and (S.2)
- **return** *p-bit states in decimal*

Function `PAOA-circuit` (N_E, J, N, p) **:**
- **for** $k \leftarrow 1$ **to** N_E **do**
  - state $\leftarrow$ `p-computer` (J, N, p)
  - save the p-bit states
- find the estimated distribution ($\hat{\boldsymbol{\rho}}_p$) using equation (S.3)
- compute the cost ($\mathcal{L}$) using equation (S.4)
- **return** *cost*

while *(step size $>$ ε_{step} and number of iterations $<$ $t_{\max}$)* **do**
- cost $\leftarrow$ PAOA-circuit(N_E, J, N, p)
- minimize cost and get a perturbation vector (p) using a gradient-free optimizer
- **for** $i \leftarrow 1$ **to** p **do**
  - $J^{(i)}_{t+1} \leftarrow J^{(i)}_t + p^{(i)}$
- $t \leftarrow t + 1$

return *optimal variational parameters*

p-computer subroutine in Algorithm.S1 uses p-bit equations to generate samples from a (J, h) parameterized distribution. In equations. (S.1) and (S.2), we used two variations of this algorithm. In the full-adder case, we set $\beta = 1$, $h = 0$, and optimize J.

$$m_i = \text{sgn}[\tanh(\beta I_i) - \text{rand}_\text{u}(-1, 1)], \tag{S.1}$$

In the majority gate problem, the graph weights and biases are set to one and zero, respectively ($J_{ij} = +1, h = 0$). Then, the annealing schedule is localized for each node (β_i), that is, each p-bit gets its own schedule.

$$I_i = \sum_j J_{ij} m_j + h_i. \tag{S.2}$$

Since the cost function is defined over the distribution of the target states, we estimated the distribution using the generated independent samples by counting the frequency of observing the desired states over N_E independent experiments (equation (S.3)). For accurate estimation, we use ten million experiments, which bounds the deviation from the true distribution to approximately 3.2×10^{-4}.

$$\hat{\boldsymbol{\rho}}^{(j)}(\{m\}) = \frac{1}{N_E} \sum_{k=1}^{N_E} \mathbb{1}\{X_{j,k} = \{m\}\}, \quad \{m\} \in \Omega \tag{S.3}$$

In the negative log-likelihood function defined in equation (S.4), the estimated distribution over a target states set $\mathcal{X}$ is used to calculate the associated loss. The variational parameters $\boldsymbol{\theta}$ represent the parameters being optimized, such as J_{ij} for the full-adder, and β_i for the majority gate problem.

$$\mathcal{L}(\boldsymbol{\theta}) = -\sum_{\{m\}\in\mathcal{X}} \ln(\hat{\boldsymbol{\rho}}_p(\{m\};\boldsymbol{\theta})), \tag{S.4}$$

1. FULL-ADDER WITH FULLY PARAMETRIZED PAOA ANSATZ

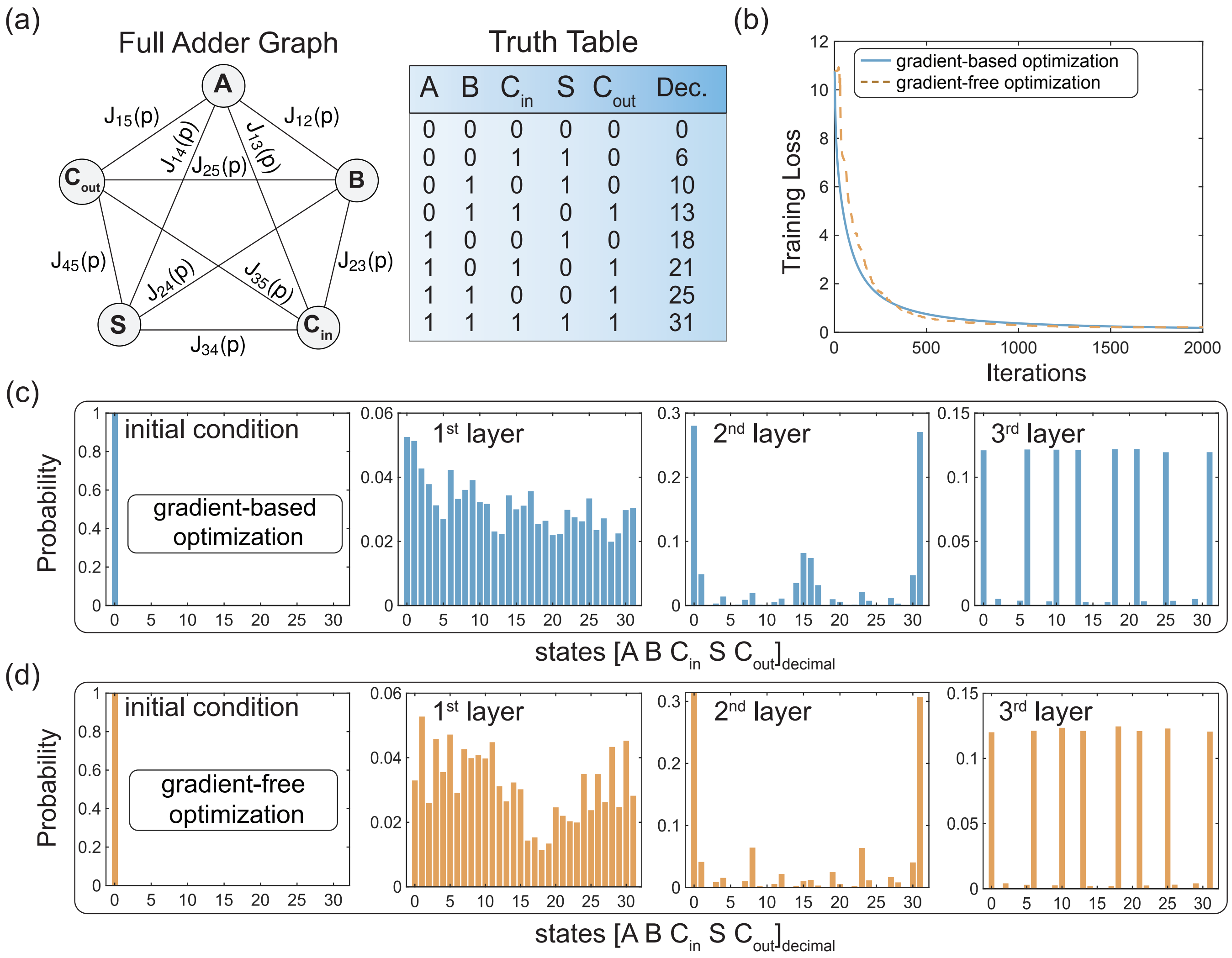

Fig. S1. (a) All-to-All full-adder network, showing graph weights $J(p)$, and truth table, where Dec. refers to the decimal representation of the state of [A B C_{in} S C_{out}] from left to right. (b) Training loss over optimization iterations for gradient-based and gradient-free methods. (c) PDF evolution across three layers, obtained from a Markov chain. (d) PDF evolution across three time layers, using MCMC (10^7 experiments).

The full-adder is represented as a fully connected network comprising five p-bits, where the graph weights are the only variational parameters (as shown in Supplementary Fig. S1a). As depicted in the same figure, the graph weights evolve with p, such that for each layer, we have different weights that are responsible for the PDF evolution. The full-adder performs 1-bit binary addition with three inputs (A, B, and Carry in = C_{in}) and two outputs (Sum = S and Carry out = C_{out}). We use a finite Markov chain with three layers (p=3), and $\boldsymbol{\rho}_0 = \begin{bmatrix}1 & 0 & 0 & 0 & 0\end{bmatrix}^\top$ as the initial condition PDF. The optimization is carried out exactly similar to the majority gate problem in Section. 2, except the parameterization here is the graph weights. The optimal parameters, rounded to the nearest hundredth, are presented in equation (S.5). The results in Supplementary Fig. S1c and Fig. S1d show clear agreement between the two methods, namely, the gradient-based approach where the probability transition matrix is constructed, and the gradient-free one where samples are generated to estimate the underlying distribution. More details on the gradient-based method are provided in Section. 3, where we show how an AND gate can be solved analytically.

$$J^{(1)}_{\text{optimal}} = \begin{pmatrix} 0 & 0.18 & 0.12 & 0 & -0.10 \\ 0.18 & 0 & -0.08 & 0.06 & -0.11 \\ 0.12 & -0.08 & 0 & 0.01 & -0.05 \\ 0 & 0.06 & 0.01 & 0 & 0.02 \\ -0.10 & -0.11 & -0.05 & 0.02 & 0 \end{pmatrix}, J^{(2)}_{\text{optimal}} = \begin{pmatrix} 0 & 0.31 & 0.43 & 1.14 & 0.17 \\ 0.31 & 0 & 0.15 & 0.58 & -0.32 \\ 0.43 & 0.15 & 0 & 1.26 & -0.28 \\ 1.14 & 0.58 & 1.26 & 0 & 1.45 \\ 0.17 & -0.32 & -0.28 & 1.45 & 0 \end{pmatrix}, J^{(3)}_{\text{optimal}} = \begin{pmatrix} 0 & -1.68 & -1.91 & 1.90 & 1.93 \\ -1.68 & 0 & -2.31 & 1.87 & 2.25 \\ -1.91 & -2.31 & 0 & 1.84 & 2.58 \\ 1.90 & 1.87 & 1.84 & 0 & -3.93 \\ 1.93 & 2.25 & 2.58 & -3.93 & 0 \end{pmatrix} \tag{S.5}$$

The simulation parameters used here are tabulated in Table S1. In gradient-based approach, the training terminates when either the tolerance in gradient, $\|\nabla\mathcal{L}(\boldsymbol{\theta})\| < \varepsilon_{\text{grad}}$, or maximum iterations is met because updates of such order no longer improve the objective. For derivative-free (Cobyla), convergence is declared when the step-size (trust–region radius) falls below a chosen tolerance $\Delta\theta < \varepsilon_{\text{step}}$, signalling that all admissible simplex moves would alter the variational parameters by a negligible change, and hence further iterations are unproductive.

TABLE S1. Full Adder Simulation Parameters.

Parameter	Value
number of nodes (N)	5
number of layers (p)	3
update order	$\{m_1, m_2, m_3, m_4, m_5\}$
initial parameters ($J^{(1)}$, $J^{(2)}$, $J^{(3)}$)	0.1
learning rate (η)	0.01
tolerance in gradient ($\varepsilon_{\text{grad}}$)	10^{-6}
maximum iterations ($t_{\max}$)	2000
number of experiments (N_E)	10^7
tolerance ($\varepsilon_{\text{step}}$)	10^{-6}

2. MAJORITY GATE PROBLEM

In this section, we present the optimal parameters obtained for the majority gate problem, solved in the Representative problem: majority gate subsection in the main text, using a local-annealing schedule ansätze with two layers ($p=2$). In this formulation, each node is assigned a private inverse temperature β, allowing the model to adaptively capture local structure in the optimization landscape. The full set of simulation parameters used to obtain these results is summarized in Table S2.

TABLE S2. Majority Gate Simulation Parameters.

Parameter	Value
number of nodes (N)	4
number of layers (p)	2
update order	$\{m_1, m_2, m_3, m_4\}$
initial parameters ($\bar{J}^{(1)}$, $\bar{J}^{(2)}$)	1
learning rate (η)	0.004
tolerance in gradient ($\varepsilon_{\text{grad}}$)	10^{-7}
maximum iterations ($t_{\max}$)	5000
number of experiments (N_E)	10^7
tolerance ($\varepsilon_{\text{step}}$)	10^{-7}

The optimal parameters for the nodes labeled $[A, B, C, Y]$, rounded to the nearest decimal, are:

$$\begin{array}{c} \\ \beta_A \\ \beta_B \\ \beta_C \\ \beta_Y \end{array} \begin{array}{c} p=1 \quad p=2 \\ \begin{bmatrix} 0.8 & -0.2 \\ 0.8 & 0 \\ 0.9 & 0 \\ 0.5 & 2.7 \end{bmatrix} \end{array} \tag{S.6}$$

These variational parameters, when combined with the initial graph weights ($J_{ij} = +1$), define the effective couplings $\bar{J}_{ij}^{(k)} = \beta_i^{(k)} J_{ij}$. The optimized parameters can be used to solve the majority gate problem (shown in Figs. 2 in the main text).

3. ANALYTICAL FORMULATION OF PAOA: AND GATE PROBLEM

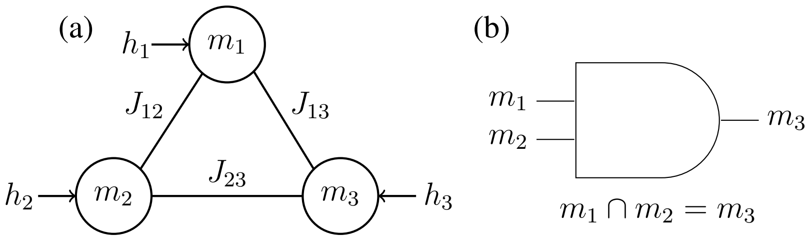

Truth Table

m_1	m_2	m_3	Dec.
0	0	0	0
0	1	0	2
1	0	0	4
1	1	1	7

Fig. S2. (a) A schematic of AND gate as an undirected graph. (b) The AND gate schematic along with the truth table, where Dec. refers to the decimal representation of the state $[m_1,m_2,m_3]$ from left to right.

In this section, we show the details of learning the weights of an AND gate analytically using the fully parameterized PAOA ansatz. The problem is shown in Supplementary Fig. S2. Using the fully-parametrized-PAOA, the J graph weight matrix and bias vector h can be written as:

$$J = \begin{pmatrix} 0 & J_{12} & J_{13} \\ J_{12} & 0 & J_{23} \\ J_{13} & J_{23} & 0 \end{pmatrix}, \quad h = \begin{bmatrix} h_1 \\ h_2 \\ h_3 \end{bmatrix} \tag{S.7}$$

For a general graph, the entries of w_k are given by

$$\begin{aligned} [w_k]_{ab} &= P(a \leftarrow b) \\ &= \begin{cases} \dfrac{1 + m_k^{(a)} \tanh(I_k^{(b)})}{2} & \text{if } m^{(a)} \text{ and } m^{(b)} \text{ are identical} \\ & \text{except possibly at the } k^{\text{th}} \text{ bit,} \\ 0 & \text{otherwise} \end{cases} \end{aligned} \tag{S.8}$$

where $I_k^{(b)} = \sum_\ell J_{k\ell} m_\ell^{(b)} + h_k$ is the synaptic input to node k based on the configuration $m^{(b)}$, and $m^{(a)}$ denotes the spin configuration after updating bit k.

Using equations (S.7) and (S.8) with the following update order $\{m_1, m_2, m_3\}$, the probability transition matrix (W) can be constructed as follows:

$$\begin{aligned} W &= w_3 w_2 w_1 \\ &= \underbrace{\begin{bmatrix} t & t & 0 & 0 & 0 & 0 & 0 & 0 \\ t' & t' & 0 & 0 & 0 & 0 & 0 & 0 \\ 0 & 0 & u & u & 0 & 0 & 0 & 0 \\ 0 & 0 & u' & u' & 0 & 0 & 0 & 0 \\ 0 & 0 & 0 & 0 & u' & u' & 0 & 0 \\ 0 & 0 & 0 & 0 & u & u & 0 & 0 \\ 0 & 0 & 0 & 0 & 0 & 0 & t' & t' \\ 0 & 0 & 0 & 0 & 0 & 0 & t & t \end{bmatrix}}_{\text{update } m_3 \text{ conditioned on } \{m_2, m_1\}} \times \underbrace{\begin{bmatrix} r & 0 & r & 0 & 0 & 0 & 0 & 0 \\ 0 & s & 0 & s & 0 & 0 & 0 & 0 \\ r' & 0 & r' & 0 & 0 & 0 & 0 & 0 \\ 0 & s' & 0 & s' & 0 & 0 & 0 & 0 \\ 0 & 0 & 0 & 0 & s' & 0 & s' & 0 \\ 0 & 0 & 0 & 0 & 0 & r' & 0 & r' \\ 0 & 0 & 0 & 0 & s & 0 & s & 0 \\ 0 & 0 & 0 & 0 & 0 & r & 0 & r \end{bmatrix}}_{\text{update } m_2 \text{ conditioned on } \{m_3, m_1\}} \times \underbrace{\begin{bmatrix} p & 0 & 0 & 0 & p & 0 & 0 & 0 \\ 0 & q & 0 & 0 & 0 & q & 0 & 0 \\ 0 & 0 & q' & 0 & 0 & 0 & q' & 0 \\ 0 & 0 & 0 & p' & 0 & 0 & 0 & p' \\ p' & 0 & 0 & 0 & p' & 0 & 0 & 0 \\ 0 & q' & 0 & 0 & 0 & q' & 0 & 0 \\ 0 & 0 & q & 0 & 0 & & q & 0 \\ 0 & 0 & 0 & p & 0 & 0 & 0 & p \end{bmatrix}}_{\text{update } m_1 \text{ conditioned on } \{m_3, m_2\}} \\ &= \begin{array}{c} \\ 000 \\ 001 \\ 010 \\ 011 \\ 100 \\ 101 \\ 110 \\ 111 \end{array} \begin{array}{c} \begin{array}{cccccccc} 000 & 001 & 010 & 011 & 100 & 101 & 110 & 111 \end{array} \\ \begin{bmatrix} prt & qst & q'rt & p'st & prt & qst & q'rt & p'st \\ prt' & qst' & q'rt' & p'st' & prt' & qst' & q'rt' & p'st' \\ pr'u & qs'u & q'r'u & p's'u & pr'u & qs'u & q'r'u & p's'u \\ pr'u' & qs'u' & q'r'u' & p's'u' & pr'u' & qs'u' & q'r'u' & p's'u' \\ p's'u' & q'r'u' & qs'u' & pr'u' & p's'u' & q'r'u' & qs'u' & pr'u' \\ p's'u & q'r'u & qs'u & pr'u & p's'u & q'r'u & qs'u & pr'u \\ p'st' & q'rt' & qst' & prt' & p'st' & q'rt' & qst' & prt' \\ p'st & q'rt & qst & prt & p'st & q'rt & qst & prt \end{bmatrix} \end{array} \end{aligned} \tag{S.9}$$

where

$$\begin{aligned}
p &= \frac{1+\tanh(J_{12}+J_{13}+h_1)}{2}, \quad q = \frac{1+\tanh(J_{12}-J_{13}+h_1)}{2}, \quad r = \frac{1+\tanh(J_{12}+J_{23}+h_2)}{2} \\
p' &= \frac{1-\tanh(J_{12}+J_{13}+h_1)}{2}, \quad q' = \frac{1-\tanh(J_{12}-J_{13}+h_1)}{2}, \quad r' = \frac{1-\tanh(J_{12}+J_{23}+h_2)}{2} \\
s &= \frac{1+\tanh(J_{12}-J_{23}+h_2)}{2}, \quad t = \frac{1+\tanh(J_{13}+J_{23}+h_3)}{2}, \quad u = \frac{1+\tanh(J_{13}-J_{23}+h_3)}{2} \\
s' &= \frac{1-\tanh(J_{12}-J_{23}+h_2)}{2}, \quad t' = \frac{1-\tanh(J_{13}+J_{23}+h_3)}{2}, \quad u' = \frac{1-\tanh(J_{13}-J_{23}+h_3)}{2}
\end{aligned} \tag{S.10}$$

Note that $x + x' = 1, \ x \in \{p, q, r, s, t, u\}$. To illustrate the procedure, let's examine the construction of following entry W_{81}. Using Gibbs sampling, we get

$$\begin{aligned}
W_{81} &= P(\{1,1,1\} \longleftarrow \{-1,-1,-1\}) \\
&= \underbrace{\frac{1+\tanh(J_{12}m_2+J_{13}m_3+h_1)}{2}}_{m_2=-1, m_3=-1} \times \underbrace{\frac{1+\tanh(J_{21}m_1+J_{23}m_3+h_2)}{2}}_{m_1=1, m_3=-1} \times \underbrace{\frac{1+\tanh(J_{31}m_1+J_{32}m_2+h_3)}{2}}_{m_1=1, m_2=1} \\
&= p'st
\end{aligned} \tag{S.11}$$

Note the use of updated value of m_1 in updating m_2. Similarly, we used the updated value of m_1 and m_2 in updating m_3. This is basically indicating that p-bits are updated sequentially. Following the same procedure, the rest of W entries can be filled. This W matrix is used now to train the AND gate with analytical derivatives.

We illustrate the procedure for training an AND gate, which can be extended in the same manner to a full-adder. The training is accomplished in three main steps:

(i) Construct the transition matrix. We define the probability transition matrix W using the coupling weights $\{J_{12}, J_{23}, J_{13}\}$ and biases $\{h_1, h_2, h_3\}$, as depicted in Supplementary Fig. S2(a). For a network with p layers, one may assign separate W (and hence distinct sets of J and h) for each layer.

(ii) Specify a loss function. We adopt the loss function given by equation (S.4), evaluated over the truth table states in Supplementary Fig. S2(b). For the AND gate, these states are $\mathcal{X} = \{(0,0,0), (0,1,0), (1,0,0), (1,1,1)\}$.

(iii) Perform gradient descent. Using gradient-descent we iteratively update each weight and bias until convergence.

For simplicity, we set $p = 1$ (a single layer), which suffices to obtain the desired states. We also choose $\boldsymbol{\rho}_0 = \begin{bmatrix} 1 & 0 & \cdots & 0 \end{bmatrix}^\top$ as the initial configuration, though in principle any initial state may be used. Notably, the final optimal parameters will be valid only for this specific initial choice, although the approach generalizes to arbitrary $\boldsymbol{\rho}_0$.

Carrying out the above steps leads to

$$\underbrace{\begin{bmatrix}
prt & qst & q'rt & p'st & prt & qst & q'rt & p'st \\
prt' & qst' & q'rt' & p'st' & prt' & qst' & q'rt' & p'st' \\
pr'u & qs'u & q'r'u & p's'u & pr'u & qs'u & q'r'u & p's'u \\
pr'u' & qs'u' & q'r'u' & p's'u' & pr'u' & qs'u' & q'r'u' & p's'u' \\
p's'u' & q'r'u' & qs'u' & pr'u' & p's'u' & q'r'u' & qs'u' & pr'u' \\
p's'u & q'r'u & qs'u & pr'u & p's'u & q'r'u & qs'u & pr'u \\
p'st' & q'rt' & qst' & prt' & p'st' & q'rt' & qst' & prt' \\
p'st & q'rt & qst & prt & p'st & q'rt & qst & prt
\end{bmatrix}}_{W}
\underbrace{\begin{bmatrix} 1 \\ 0 \\ 0 \\ 0 \\ 0 \\ 0 \\ 0 \\ 0 \end{bmatrix}}_{\boldsymbol{\rho}_0}
=
\underbrace{\begin{bmatrix} prt \\ prt' \\ pr'u \\ pr'u' \\ p's'u' \\ p's'u \\ p'st' \\ p'st \end{bmatrix}}_{\boldsymbol{\rho}_p},
\tag{S.12}$$

where $\boldsymbol{\rho}_p$ then enters the loss function as follows:

$$\begin{aligned}
\mathcal{L}(\boldsymbol{\theta}) &= -\sum_{\{m\}\in\mathcal{X}} \ln\big[\boldsymbol{\rho}_p(\{m\};\boldsymbol{\theta})\big] \\
&= -\Big[\ln\big(prt\big) + \ln\big(pr'u\big) + \ln\big(ps'u'\big) + \ln\big(p'st\big)\Big],
\end{aligned} \tag{S.13}$$

with parameter vector $\boldsymbol{\theta} = \begin{bmatrix} J_{12} & J_{23} & J_{13} & h_1 & h_2 & h_3 \end{bmatrix}^\top$.

Its derivatives with respect to each parameter are given by:

$$\frac{\partial \mathcal{L}}{\partial J_{12}} = \operatorname{sech}\big(h_2 + J_{12}\big)\Big\{-\operatorname{sech}\big(h_1 + J_{12} + J_{13}\big)\Big[\cosh\big(h_1 - h_2 + J_{13}\big) + \cosh\big(h_1 + h_2 + 2\,J_{12} + J_{13}\big) - 4\,\sinh\big(h_1 + h_2 + 2\,J_{12} + J_{13}\big)\Big] + 2\,\sinh\big(J_{23}\big)\Big[-\operatorname{sech}\big(h_2 + J_{12} - J_{23}\big) + \operatorname{sech}\big(h_2 + J_{12} + J_{23}\big)\Big]\Big\}$$

$$\frac{\partial \mathcal{L}}{\partial J_{13}} = 2\operatorname{sech}\big(h_3 + J_{13}\big)\Big\{\sinh(J_{23})\big(\operatorname{sech}[\,h_3 + J_{13} + J_{23}\,] - \operatorname{sech}[\,h_3 + J_{13} - J_{23}\,]\big) - \operatorname{sech}\big(h_1 + J_{12} + J_{13}\big)\Big(\cosh\big(h_1 - h_3 + J_{12}\big) + \cosh\big(h_1 + h_3 + J_{12} + 2\,J_{13}\big) - 2\,\sinh\big(h_1 + h_3 + J_{12} + 2\,J_{13}\big)\Big)\Big\}$$

$$\frac{\partial \mathcal{L}}{\partial J_{23}} = -2\tanh\big(h_2 + J_{12} - J_{23}\big) - 2\tanh\big(h_3 + J_{13} - J_{23}\big) + 2\tanh\big(h_2 + J_{12} + J_{23}\big) + 2\tanh\big(h_3 + J_{13} + J_{23}\big) - 2$$

$$\frac{\partial \mathcal{L}}{\partial h_1} = 4\tanh\big(h_1 + J_{12} + J_{13}\big) - 2$$

$$\frac{\partial \mathcal{L}}{\partial h_2} = 2\,\operatorname{sech}\big(h_2 + J_{12} - J_{23}\big)\operatorname{sech}\big(h_2 + J_{12} + J_{23}\big)\sinh\big(2(h_2 + J_{12})\big)$$

$$\frac{\partial \mathcal{L}}{\partial h_3} = -\operatorname{sech}\big(h_3 + J_{13} - J_{23}\big)\operatorname{sech}\big(h_3 + J_{13} + J_{23}\big)\Big[\cosh\big(2\,(h_3 + J_{13})\big) + \cosh\big(2\,J_{23}\big) - 2\,\sinh\big(2\,(h_3 + J_{13})\big)\Big]$$

Finally, the gradient-descent update rules are:

$$\begin{aligned} J_{ij}^{(t+1)} &\leftarrow J_{ij}^{(t)} - \eta\,\frac{\partial \mathcal{L}}{\partial J_{ij}^{(t)}}, \quad 1 \le i < j \le 3, \\ h_i^{(t+1)} &\leftarrow h_i^{(t)} - \eta\,\frac{\partial \mathcal{L}}{\partial h_i^{(t)}}, \quad 1 \le i \le 3, \end{aligned} \tag{S.15}$$

where η is a hyperparameter (step size). Each iteration refines the parameters and thus updates the transition matrix W, yielding the new distribution $\boldsymbol{\rho}_p^{(t+1)}$. The stopping criterion is set to be either a fixed iteration budget or a certain tolerance in the gradient.

Using the method explained above with $\eta = 0.02$, maximum iterations of 2000, tolerance in the gradient of 10^{-6}, and uniform random initialization of weights and biases, specifically, $\boldsymbol{\theta} \sim \mathrm{rand_u}[-0.5, 0.5]$, we get the following rounded optimal weights and biases:

$$J_{\text{optimal}} = \begin{pmatrix} 0 & 0 & 2.25 \\ 0 & 0 & 2.25 \\ 2.25 & 2.25 & 0 \end{pmatrix}, \quad h_{\text{optimal}} = \begin{bmatrix} 2.25 \\ 2.25 \\ -2.25 \end{bmatrix} \tag{S.16}$$

The training loss across optimization iterations and the final distribution generated using the optimal parameters are shown in Supplementary Fig. S2.

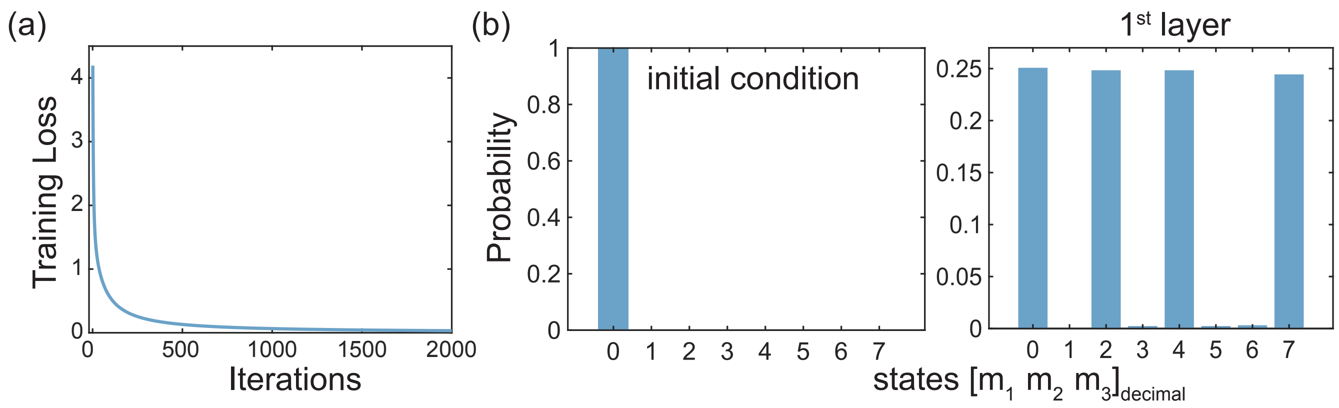

Fig. S3. (a) Cost versus optimization iterations for the probabilistic AND-gate, trained using the negative log-likelihood and gradient descent. (b) The system PDF evolution using the optimal weights and biases.

4. FPGA IMPLEMENTATION OF ON-CHIP ANNEALING

In the main paper, we present experimental results obtained using a hybrid classical-probabilistic computing system. Here, we provide the technical details of the FPGA-based p-computer implemented on the Xilinx VCU128 data center accelerator card.

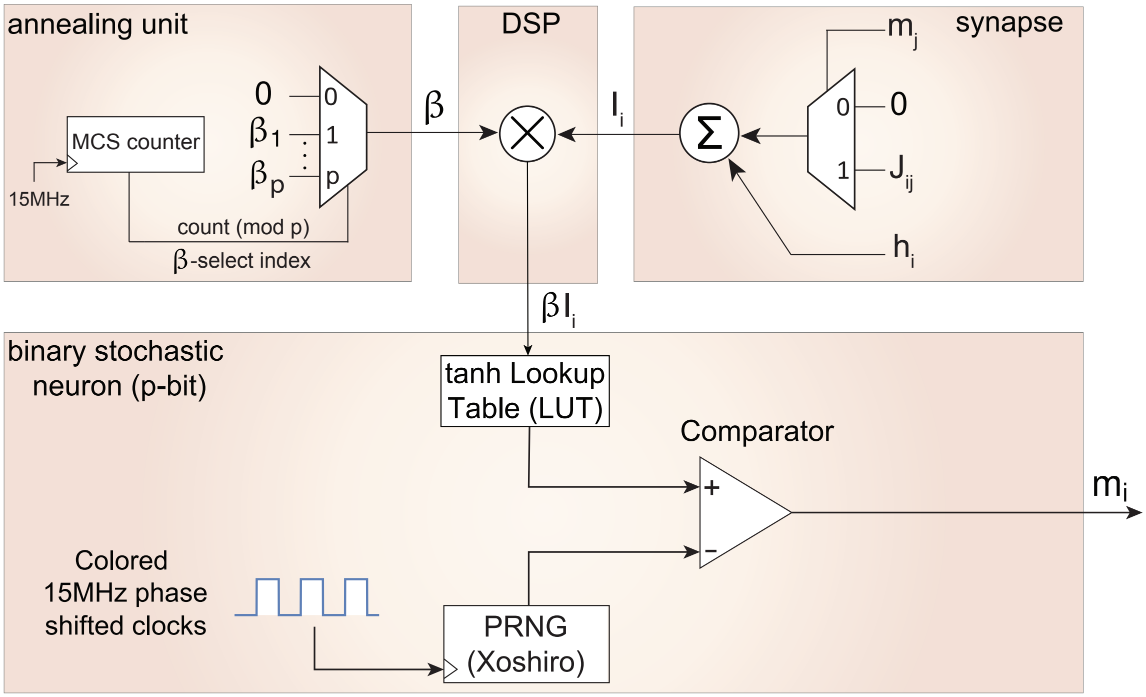

Fig. S4. Probabilistic computer architecture with on-chip annealing. The annealing unit is a $p \times 1$ multiplexer controlled by a counter that updates β value after MCSs budget is elapsed. The synapse block implements equation (S.2) by using a 2×1 multiplexer and finding the sum over all states $\{m\}$. The DSP slice carries out the multiplication of β and the synapse input I_i. The BSN unit implements equation (S.1), uses lookup table for tanh, Xoshiro [1] as the pseudorandom number generator, and a comparator to update the p-bit states.

The p-computer architecture with on-chip annealing (shown in Supplementary Fig. S4) consists of four main blocks as follows:

- Annealing unit: outputs β based on the MCS counter. The MCS counter increases once a fixed MCSs budget is elapsed. The value of the counter is then used to select the value of the β. After exhausting all β values for all layers, the counter value will start from the beginning for a new experiment. For the experiments to be independent, the initial β value is set to zero to randomize the p-bits. The fixed-point precision used here is $s\{4\}\{5\}$, where s denotes the signed bit, and the values in the square brackets represent the integer and fraction bits, respectively.
- Synapse: outputs I_i based on the states, weights, and biases. For each m_j, the multiplexer either chooses J_{ij} or zero. All results are then added at the end along with the bias to calculate the input to node i. The fixed-point precision used here for weights and biases is $s\{4\}\{5\}$.
- DSP: outputs the multiplication of I_i and β.
- Binary stochastic neuron: outputs the updated binary state of node i, m_i. The output of the DSP block is taken to the LUT to find the corresponding $\tanh$ value. This value is compared with a random number generated via Xoshiro [1] using a comparator.

5. PARAMETERS TRAINED ON SMALL-SIZE SK INSTANCES FOR LARGER PROBLEMS

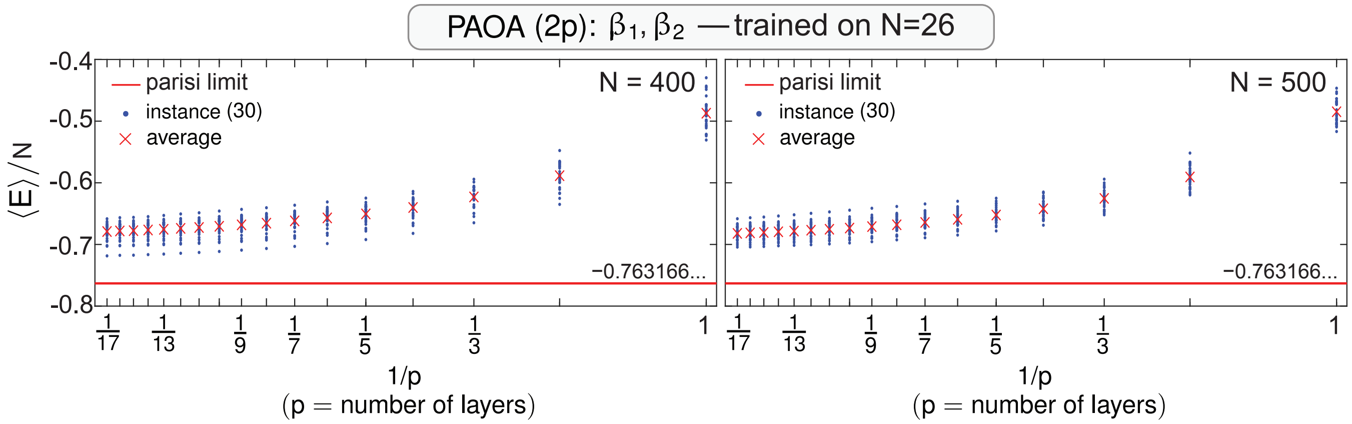

Fig. S5. The average energy per spin $\langle E\rangle/N$ for 30 random large SK instances of size $N \in \{400, 500\}$ using parameters trained on N=26 and 10^6 independent experiments.

In this section, we evaluate the generalization of parameters trained on N = 26-spin SK instances by applying them to significantly larger problems with N = 400 and N = 500, without any additional fine-tuning. Fig. S5 shows the average energy per spin for these larger Sherrington–Kirkpatrick systems, plotted alongside the Parisi value, which represents the ground state energy in the thermodynamic limit. As the number of PAOA layers increases, the average energy decreases and the instance-to-instance variation narrows and concentrates-more so than in the N = 26 case. This behavior suggests that the optimized schedules generalize effectively to larger system sizes, and that the remaining fluctuations are primarily due to finite-size effects. It is worth noting that problem sizes of this scale are currently intractable for existing QAOA hardware, underscoring a key practical advantage of PAOA.

6. METHODOLOGY OF SCHEDULE PRIVATIZATION IN THE LÉVY SK MODEL

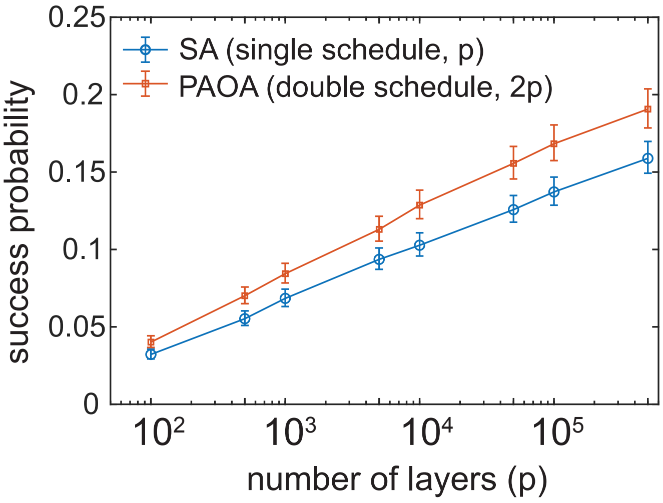

Fig. S6. Average success probability over 500 instances comparing single-schedule SA (blue) and double-schedule PAOA (red) for N=50 and a 50%-50% split. Error bars are 95% confidence intervals from 10^5 bootstrap samples with replacement.

To evaluate the performance difference between conventional single-schedule simulated annealing and a multi-schedule annealing scheme applied to the SK model with Lévy bonds [2], the following two-step procedure is adopted:

1. Baseline schedule optimization. We perform a grid search over final inverse-temperature values to identify an optimal single geometric annealing schedule at shallow depth (p=17). The initial inverse temperature (corresponding to high temperature) is held fixed at a small value, while the final value is varied to minimize the average energy per spin across multiple instances and multiple runs.

2. Privatized schedule generation. Using the optimized schedule from step 1, denoted as β_1, we introduce schedule heterogeneity by applying a multiplicative spacing factor Δ, which is guided by the separation exhibited by PAOA trained double schedules. The original β_1 is assigned to heavy nodes (i.e., those strongly coupled), while the light nodes receive a schedule scaled by $(1+\Delta)$, i.e., $\beta_2 = (1+\Delta)\beta_1$, where Δ is chosen to reflect the separation that shallow PAOA averaged schedule exhibited when trained on N=50 with 50 instances in (see Fig.5b in the main text). We note that the split -used in Fig.5 in the main text- into heavy and light groups was done such that top 20% (heaviest 10 nodes) are assigned β_1, and the rest is assigned β_2. As shown in Fig. S6, when using a 50%-50% split, we observe similar behavior shown in Fig. 5d in the main text.

This procedure follows closely the guidance of PAOA, which suggests scaling the optimized single schedule up and assigning it to the light nodes rather than scaling down the best single annealing schedule and assigning it to the heavy nodes, while keeping the light nodes running at the best annealing schedule. This choice is determined solely by PAOA owing to its learning capability.

The impact of this scheduling strategy is assessed by comparing the success probability of solving 500 instances, showing that the double schedules learned by PAOA have a consistent and a statistical advantage over a single annealing schedule.

REFERENCES

[1] David Blackman and Sebastiano Vigna. Scrambled linear pseudorandom number generators. *ACM Trans. Math. Softw.*, 47(4), September 2021. ISSN 0098-3500.

[2] Stefan Boettcher. Ground states of the sherrington–kirkpatrick spin glass with levy bonds. *Philosophical Magazine*, 92(1-3):34–49, 2012.
